# Predictive factors for postoperative recurrence in early cervical cancer patients: a meta-analysis

**DOI:** 10.3389/fsurg.2025.1588558

**Published:** 2025-06-19

**Authors:** Weili Hou, Yaru Ma, Suli Sun, Yanlei Gao, Jia Ling, Rui Shi

**Affiliations:** ^1^ Maternity Services Center, Shijiazhuang Maternity & Child Healthcare Hospital, Shijiazhuang, China; ^2^ Gynaecological Clinic, Shijiazhuang Maternity & Child Healthcare Hospital, Shijiazhuang, China; ^3^ Department of Medical Imaging, Shijiazhuang Maternity & Child Healthcare Hospital, Shijiazhuang, China; ^4^ Department of Gynecology, Shijiazhuang Maternity & Child Healthcare Hospital, Shijiazhuang, China

**Keywords:** recurrence risk, prognostic factors, meta-analysis, tumor characteristics, early-stage cervical cancer

## Abstract

**Background:**

Early-stage cervical cancer generally has a favorable prognosis with treatment, yet recurrence remains a significant risk for a subset of patients. Identifying reliable prognostic factors can help refine risk stratification, optimize follow-up strategies, and guide adjuvant therapy decisions. This meta-analysis evaluates the association between key prognostic factors—tumor diameter, HPV status, depth of invasion, LVSI status, and positive lymph nodes—and recurrence risk in early-stage cervical cancer.

**Materials and methods:**

A systematic search of PubMed, Embase, Cochrane Library, and Scopus was conducted to identify studies assessing the relationship between prognostic factors and recurrence in early-stage cervical cancer. Studies meeting predefined inclusion criteria were selected, and data were extracted on patient demographics, tumor characteristics, and recurrence outcomes. The NOS was used to assess study quality. Pooled ORs with 95% CIs were calculated using both fixed-effects and random-effects models, with heterogeneity and publication bias assessed through I² statistics and funnel plot analysis, respectively.

**Results:**

A total of 10 studies were included in the meta-analysis. Tumor diameter >4 cm (OR = 2.49; 95% CI: 1.69–3.69), depth of invasion >1/2 (OR = 2.82; 95% CI: 1.66–4.80), LVSI positivity (OR = 2.54; 95% CI: 1.36–4.73), and positive lymph nodes (OR = 2.86; 95% CI: 1.99–4.11) were all significantly associated with an increased risk of recurrence. However, HPV-positive status showed no consistent association with recurrence risk (OR = 2.12; 95% CI: 0.31–14.52), likely due to high heterogeneity among the studies (I² = 86%). Sensitivity analyses confirmed the robustness of the results, and publication bias was minimal.

**Conclusion:**

This meta-analysis identified tumor diameter >4 cm, depth of invasion >1/2, LVSI positivity, and positive lymph nodes as significant prognostic factors for recurrence in early-stage cervical cancer. These findings underscore the importance of comprehensive assessment in clinical practice to better identify high-risk patients who may benefit from intensified monitoring or adjuvant therapies. Further research, particularly on the role of HPV status, is needed to enhance the predictive accuracy of recurrence risk models.

**Systematic Review Registration:**

https://www.crd.york.ac.uk/PROSPERO/view/CRD42024599867, PROSPERO CRD42024599867.

## 1 Introduction

Cervical cancer is one of the most common malignancies affecting women worldwide, particularly in low- and middle-income countries where access to screening and preventive measures is often limited (1, 2). According to recent global estimates, cervical cancer accounts for approximately 600,000 new cases and 340,000 deaths annually, highlighting the critical need for effective management strategies to improve patient outcomes (3). Early-stage cervical cancer, typically classified as FIGO stages IA and IB, has a relatively favorable prognosis when treated with surgery or radiotherapy (4–6). However, recurrence remains a significant concern, affecting 10%–20% of patients even after initial treatment (7). Identifying reliable prognostic factors is therefore essential for improving patient stratification and tailoring follow-up and treatment strategies.

Multiple factors have been studied for their prognostic value in predicting recurrence in early-stage cervical cancer. Among these, tumor diameter, HPV status, depth of invasion, lymph vascular space invasion (LVSI) status, and lymph node involvement have emerged as potential predictors of recurrence risk (8–10). Tumor diameter >4 cm, for example, has been associated with poorer outcomes due to the potential for increased metastatic spread (10, 11), while HPV status—particularly high-risk HPV subtypes—is thought to influence tumor aggressiveness and response to treatment (8, 12, 13). Similarly, greater depth of invasion, LVSI positivity, and lymph node metastasis have been linked to higher recurrence rates, suggesting that these factors may be valuable for risk assessment in clinical practice (14–17).

While individual studies have provided insights into these associations, the results are often inconsistent, and the effect sizes vary. Meta-analysis provides a powerful tool to combine data from multiple studies, increasing statistical power and enabling a more precise estimation of the associations between prognostic factors and recurrence risk. Therefore, this systematic review and meta-analysis aims to synthesize the available evidence on the prognostic value of tumor diameter, HPV status, depth of invasion, LVSI status, and positive lymph nodes in predicting recurrence in early-stage cervical cancer. By identifying and quantifying these relationships, this study seeks to provide a clearer understanding of key prognostic factors, contributing to improved risk stratification and potentially informing individualized patient management strategies.

## 2 Methods

### 2.1 Study design and objective

This study is a systematic review and meta-analysis, conducted in accordance with the Preferred Reporting Items for Systematic Reviews and Meta-Analyses (PRISMA) 2020 guidelines, aimed to investigate the association between various prognostic factors and recurrence risk in early-stage cervical cancer patients. The analysis focuses on five key outcomes: tumor diameter (>4 cm), HPV positive, depth of invasion (>1/2), LVSI status, and positive lymph nodes. By synthesizing data from existing studies, this meta-analysis aims to quantify the impact of these factors on patient prognosis, providing valuable insights for clinical decision-making and risk stratification. The study was registered in the International Prospective Register of Systematic Reviews (PROSPERO) with the registration number CRD 42024599867.

### 2.2 Search strategy

A comprehensive literature search was conducted to identify relevant studies examining the association between prognostic factors and recurrence risk in early-stage cervical cancer. The research included studies published up to December 2024, and was carried out across multiple databases: PubMed, Embase, Cochrane Library and Scopus. The search strategy combined terms related to cervical cancer (“cervical cancer,” “cervical carcinoma”), prognostic factors (“tumor diameter,” “HPV status,” “depth of invasion,” “LVSI status,” “positive lymph nodes”), and outcomes (“recurrence,” “relapse,” “prognosis”). Boolean operators (AND, OR) were employed to broaden or narrow the search as needed, and Medical Subject Headings (MeSH) terms were applied where applicable.

### 2.3 Eligibility criteria

Studies were included in this meta-analysis based on predefined eligibility criteria to ensure the relevance and rigor of the analysis. The inclusion criteria were as follows: (1) Population: Studies involving patients diagnosed with early-stage cervical cancer, specifically those categorized as FIGO stage IA1, IA2, IB1, and IB2; (2) Intervention/Exposure: Studies that reported at least one of the following prognostic factors—tumor diameter (>4 cm), HPV status, depth of invasion (>1/2), LVSI status, or positive lymph nodes; (3) Outcomes: Studies that assessed recurrence with results reported as odds ratios (OR) with 95% confidence intervals (CI); (4) Study Design: Cohort studies, case-control studies, or other observational studies that provided original data on the association between the specified prognostic factors and clinical outcomes of interest; and (5) Language and Publication Status: No language restrictions were applied, and only published studies and articles available in full text were included.

Exclusion criteria were as follows: (1) Studies lacking sufficient data to extract or calculate Ors or RRs; (2) Reviews, meta-analyses, conference abstracts, case reports, animal studies, and non-human studies; (3) Duplicate publications, with only the most comprehensive and recent report included.

### 2.4 Data extraction

Data extraction was conducted independently by two reviewers to ensure accuracy and consistency. For each eligible study, the following data were collected: (1) Basic Study Information: author names, year of publication, and country or region where the study was conducted; (2) Patient Characteristics: sample size, age of participants (mean or median), and relevant clinical characteristics; (3) Prognostic Factors: tumor diameter (>4 cm), HPV status, depth of invasion (>1/2), LVSI status, and positive lymph node status; (4) Outcomes: associations between prognostic factors and recurrence risk. Any discrepancies between the two reviewers in data extraction were resolved through discussion and, if necessary, consultation with a third reviewer. All extracted data were entered into a standardized spreadsheet to facilitate subsequent statistical analysis.

### 2.5 Quality assessment

The quality of the studies included was assessed independently by two reviewers using the Newcastle-Ottawa Scale (NOS), which is a widely accepted tool for evaluating the quality of observational studies in meta-analyses. The NOS assesses study quality based on three main domains: selection of study groups, comparability of groups, and ascertainment of outcomes. Each study was assigned a score ranging from 0 to 9, with higher scores indicating higher methodological quality. Specifically, studies scoring 7 points or above were classified as high quality, those scoring 5–6 points as moderate quality, and those scoring below 5 points as low quality. Discrepancies in quality assessment scores between the reviewers were resolved through discussion, and if a consensus could not be reached, a third reviewer was consulted. The results of the quality assessment were recorded in a standardized table and were used to conduct sensitivity analyses to assess the robustness of the meta-analysis results.

### 2.6 Statistical analysis

Statistical analyses were performed using the meta package in R4.3.2 software (R Foundation for Statistical Computing, Vienna, Austria). For each prognostic factor (tumor diameter >4 cm, HPV status, depth of invasion >1/2, LVSI status, and positive lymph nodes), ORs with 95% CIs were calculated to quantify the association between the factor and recurrence risk in early-stage cervical cancer. Heterogeneity was assessed using Cochran's *Q* test and the I² statistic, with an I² value greater than 50% indicating substantial heterogeneity. When heterogeneity was low (I² ≤ 50%), a fixed-effects model was applied; when heterogeneity was substantial (I² > 50%), a random-effects model was used (18). Sensitivity analyses were conducted using a leave-one-out approach, in which each included study was sequentially removed to assess the robustness of the pooled results. Due to the limited number of studies (<10) included in each meta-analysis, assessment of publication bias was not formally conducted, as recommended by the Cochrane Handbook for Systematic Reviews of Interventions (19). All statistical tests were two-sided, with a *p*-value <0.05 considered statistically significant.

### 2.7 Certainty of evidence assessment

The certainty of evidence for each pooled outcome was assessed using the Grading of Recommendations Assessment, Development, and Evaluation (GRADE) approach. Factors considered included risk of bias, inconsistency, indirectness, imprecision, and publication bias. The overall certainty of evidence was categorized as high, moderate, low, or very low.

## 3 Results

### 3.1 Study selection and characteristics

**Figure 1:**
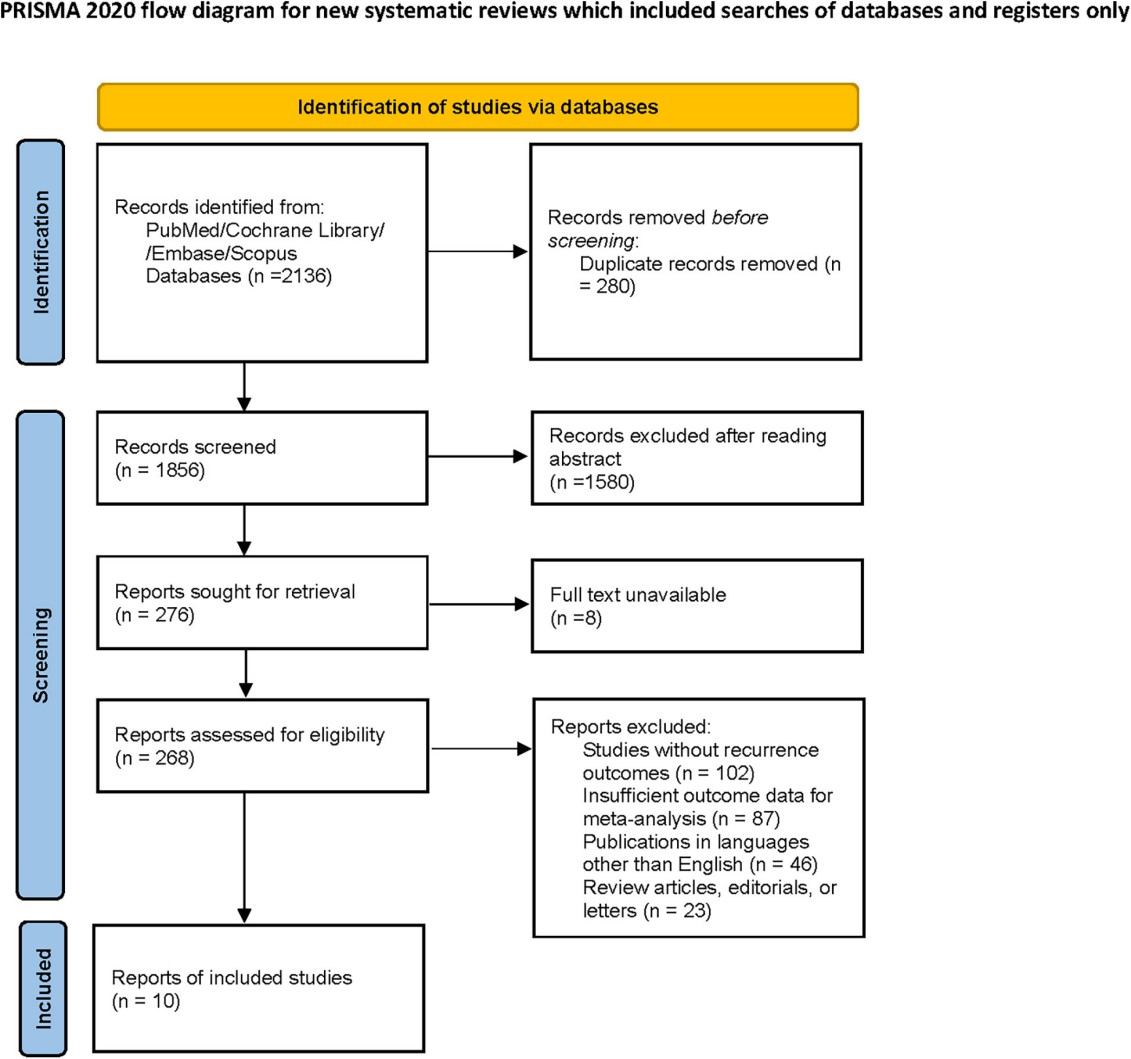
PRISMA 2020 flow diagram illustrating the study selection process for the meta-analysis.

A total of 2,136 studies were initially identified through the comprehensive database search and manual screening of reference lists. After removing duplicates, 1,856 studies were assessed for eligibility based on titles and abstracts. Subsequently, 276 full-text articles were reviewed, resulting in 10 studies being included in the final meta-analysis (Figure 1). The included studies consisted of 9 single-center studies and 1 multi-institutional study. Details on sample sizes, age distributions, tumor sizes, FIGO staging, and follow-up times are summarized in Table 1.

**Table 1:** General information of the included literature.

Author/year	Country/region	Reach type	Cases	Age (years)	Tumor size (mm), median (IQR)	FIGO stage (2009), *n* (%)				Follow-up
						IA1	IA2	IB1	IB2	
Gerdin, E. 1994 (10)	Sweden	Single-center study	167	>25	17 (10–25)	/	159 (95.21)	8 (4.79)	/	5 years
Maneo, A. 1999 (16)	Italy	Single-center study	34	51 (21–72)	> 4 cm,19 (56%), ≤4 cm, 15 (44%)	/	29 (85)	4 (12)	1 (3)	81 months (33–192 months)
Serati, M. 2012 (17)	Italy	Single-center study	282	33 (18–73)	/	/	72 (25.5)	89 (31.6)	94 (33.3)	26.7 months (6–100months)
Singh, P. 2012 (32)	Australia.	Single-center study	196	>18	/	5 (2.55%)	8 (4.08)	126 (64.29)	52 (26.53)	43.5 months (5–200 months)
Wang, J. 2015 (33)	China	Single-center study	284	>18	> 4 cm, 134 (47.2%), ≤4 cm, 150 (52.8%)	10 (3.5)	88 (30.1)	47 (16.6)	140 (49.3)	48 months
Huang, B. 2016 (11)	China	Single-center study	643	>18	/	60 (9.33)	77 (11.98)	487 (75.74)	19 (2.95)	37 months (11–97 months)
Kwon, J. 2018 (14)	Korean	Single-center study	259	47 (25–74)	/	/	57 (22.0)	202 (78.0)	/	70 months (2.5- 468.5 months)
Casarin, J. 2020 (9)	Italy/Switzerland	Multi-institutional study	428	45 (39.0–55.1)	17 (10–25)	39 (9.2)	31 (7.2)	358 (83.6)	/	56 months (1–162months)
Bae, B. K. 2022 (8)	Korea	Single-center study	252	45 (39–52)	17 (10–25)	72 (27.4)	58 (22.1)	51 (19.4)	71 (27.0%)	70.4 months (6.2–252.5 months)
Ma, G. F. 2024 (15)	China	Single-center study	310	>18	>4 cm: 123 (39.7%), ≤4 cm: 187 (60.3%)	26 (8.4%)	109 (31.9%)	91 (33.1%)	109 (31.9%)	46 months (5.7–119.4 months

IQR, interquartile range; FIGO, International Federation of Gynecology and Obstetrics; LVSI, lymphovascular space invasion; HPV, human papillomavirus.

### 3.2 Quality assessment

Quality assessment of the included studies was performed using the NOS. Scores ranged from 5 to 8, with most studies scoring 7 or above, indicating generally good methodological quality (Table 2).

**Table 2:** Literature quality assessment (NOS).

Author/year	Country/region	Selectivity (4 points)	Comparability (2 points)	outcome (3 points)	Total score (9 points)
Gerdin, E. 1994 (10)	Sweden	⋆⋆⋆⋆	⋆⋆	⋆	7
Maneo, A. 1999 (16)	Italy	⋆⋆⋆⋆	⋆	⋆	6
Serati, M. 2012 (17)	Italy	⋆⋆⋆⋆	⋆⋆	⋆	7
Singh, P. 2012 (32)	Australia.	⋆⋆⋆	⋆⋆	⋆	5
Wang, J. 2015 (33)	China	⋆⋆⋆⋆	⋆	⋆⋆	7
Huang, B. 2016 (11)	China	⋆⋆⋆⋆	⋆	⋆⋆	7
Kwon, J. 2018 (14)	Korean	⋆⋆⋆⋆	⋆	⋆⋆	6
Casarin, J. 2020 (9)	Italy/Switzerland	⋆⋆⋆⋆	⋆⋆	⋆⋆	8
Bae, B. K. 2022 (8)	Korea	⋆⋆⋆	⋆⋆	⋆⋆⋆	8
Ma, G. F. 2024 (15)	China	⋆⋆⋆⋆	⋆⋆	⋆⋆	8

### 3.3 Meta-analysis results

#### 3.3.1 Tumor diameter (>4 cm)

**Figure 2:**
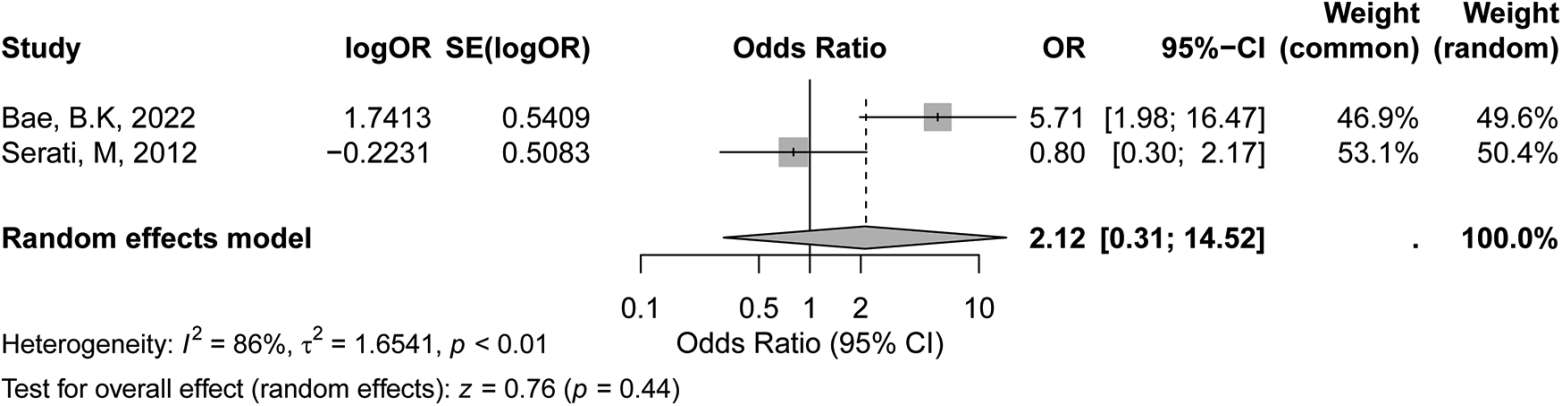
Forest plot of the association between tumor diameter (>4 cm) and recurrence risk in early-stage cervical cancer.

The meta-analysis of tumor diameter (>4 cm) included six studies, as shown in the forest plot. Based on the GRADE approach, the certainty of evidence for the association between tumor diameter >4 cm and recurrence risk were rated as moderate. Under the fixed-effects model, the pooled OR for tumor diameter greater than 4 cm was 2.16 (95% CI: 1.72–2.71, *p* < 0.01), indicating a statistically significant association between larger tumor size and increased recurrence risk in early-stage cervical cancer. Heterogeneity among the studies was moderate, with an *I²* value of 47% (*p* = 0.09) (Figure 2), suggesting some variability in effect sizes across studies, though it was not statistically significant.

#### 3.3.2 HPV positive

**Figure 3:**
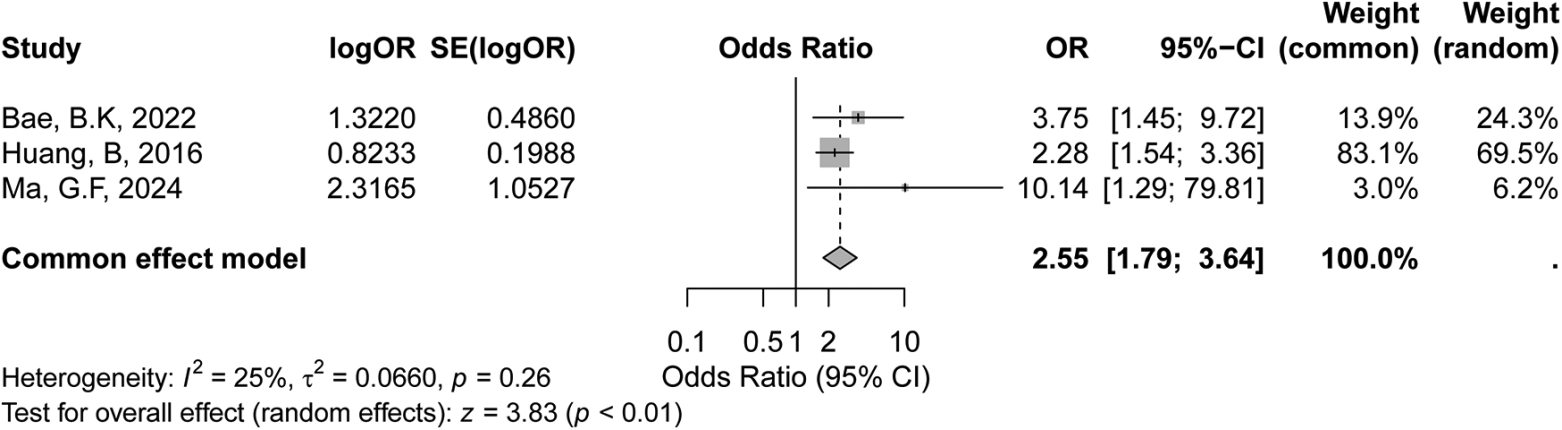
Forest plot of the meta-analysis evaluating the association between HPV-positive status and recurrence risk in early-stage cervical cancer.

The meta-analysis of HPV-positive status included two studies, as shown in the forest plot. According to the GRADE assessment, the certainty of evidence for the association between HPV-positive status and recurrence risk was rated as low, mainly due to the small number of studies and substantial heterogeneity. In the fixed-effects model, the pooled OR was 2.01 (95% CI: 0.97–4.15, *p* = 0.06), suggesting a trend towards an association between HPV positivity and increased recurrence risk, though it did not reach statistical significance. Under the random-effects model, the pooled OR was 2.12 (95% CI: 0.31–14.52, *p* = 0.44), indicating no statistically significant association between HPV-positive status and recurrence in early-stage cervical cancer. The heterogeneity across studies was high, with an I² value of 86% (*p* < 0.01) (Figure 3), indicating substantial variability in effect sizes between studies. Several factors may account for the observed inconsistency. First, the limited number of included studies reduced the statistical power. Second, variations in HPV detection methods (e.g., PCR assays, genotyping panels) and definitions of positivity (single high-risk vs. multiple HPV types) likely contributed to heterogeneity. Third, differences in patient populations, including HPV prevalence and immune response profiles, may have independently affected recurrence risk. Additionally, inconsistencies in reporting adjuvant treatments among HPV-positive patients further complicated the results.

#### 3.3.3 Depth of invasion (>1/2)

**Figure 4:**
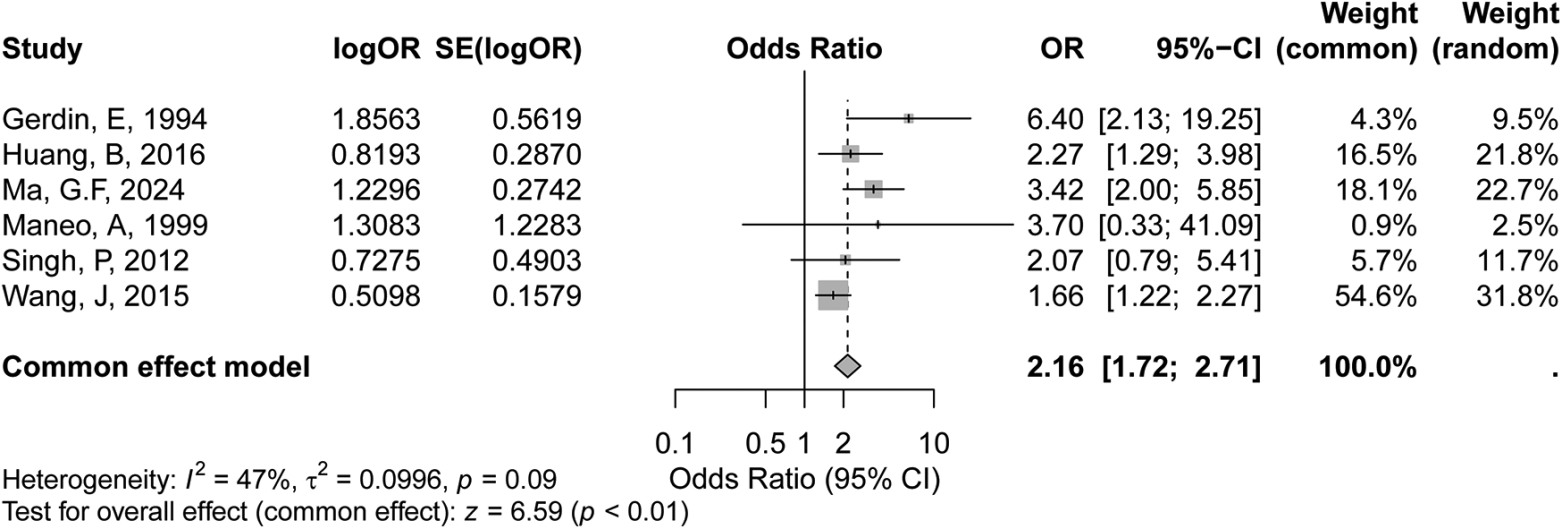
Forest plot of the meta-analysis evaluating the association between depth of invasion >1/2 and recurrence risk in early-stage cervical cancer.

The meta-analysis of depth of invasion greater than half of the cervical wall included three studies, as shown in the forest plot. The certainty of evidence for the association between depth of invasion >1/2 and recurrence risk was rated as moderate according to the GRADE criteria. In the fixed-effects model, the pooled OR was 2.55 (95% CI: 1.79–3.64, *p* < 0.01), indicating a statistically significant association between deeper invasion (>1/2) and increased recurrence risk in early-stage cervical cancer. (Figure 4), suggesting limited variability in effect sizes across studies. Overall, these findings suggest that the depth of invasion >1/2 may be a significant prognostic factor for recurrence in early-stage cervical cancer.

#### 3.3.4 LVSI positive

**Figure 5:**
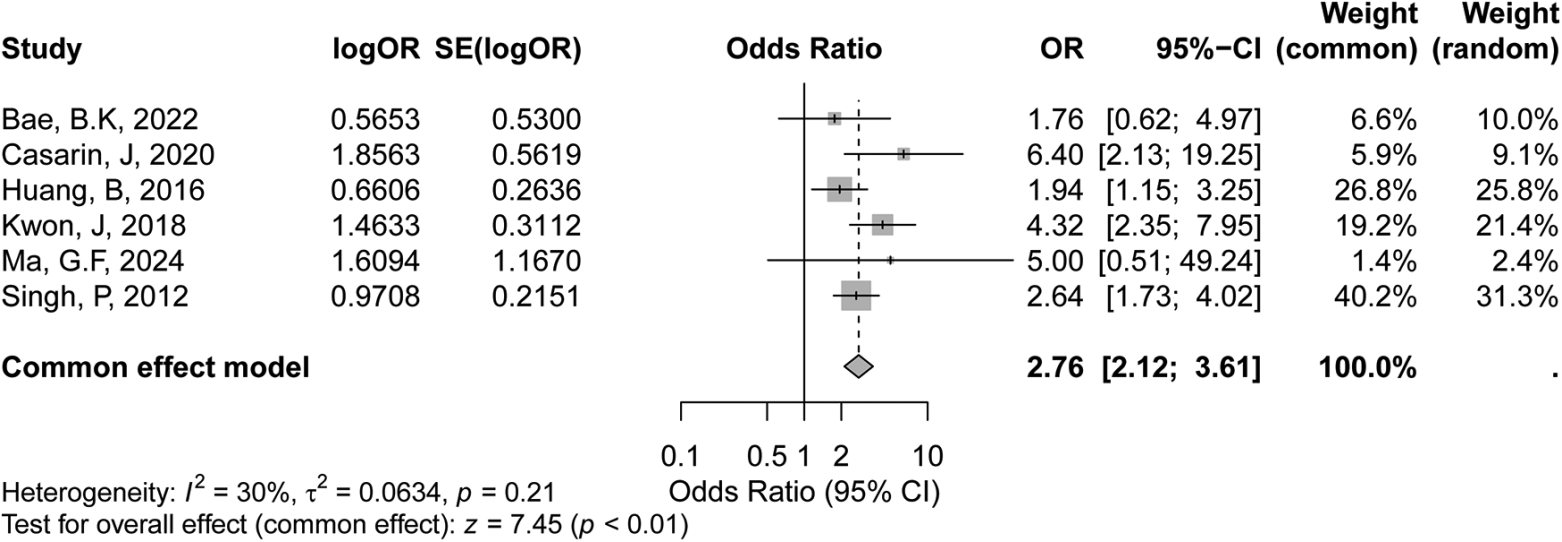
Forest plot of the meta-analysis evaluating the association between LVSI-positive status and recurrence risk in early-stage cervical cancer.

The meta-analysis evaluated the association between LVSI positive status and recurrence risk in early-stage cervical cancer included six studies, as shown in the forest plot. Based on the GRADE evaluation, the certainty of evidence for the association between LVSI positivity and recurrence risk was considered low, due to high heterogeneity among the included studies. The random-effects model yielded a similar pooled OR of 2.54 (95% CI: 1.36–4.73, *p* < 0.01), further supporting this association. However, substantial heterogeneity was observed across the studies, with an I² value of 81% (*p* < 0.01) (Figure 5), indicating significant variability in effect sizes. The high heterogeneity suggests that differences in study populations, methods, or definitions of LVSI positivity may influence the results.

#### 3.3.5 Positive lymph nodes

**Figure 6:**
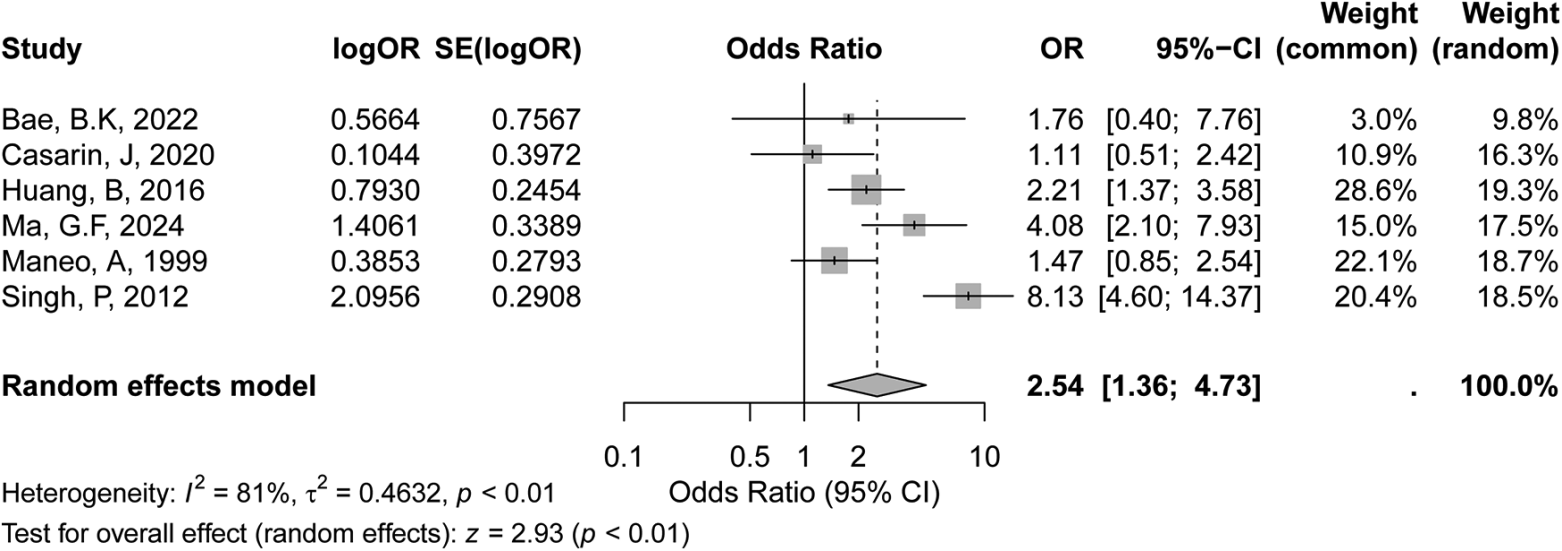
Forest plot of the meta-analysis evaluating the association between positive lymph node status and recurrence risk in early-stage cervical cancer.

The meta-analysis evaluating the association between positive lymph node status and recurrence risk in early-stage cervical cancer included six studies, as shown in the forest plot. According to the GRADE assessment, the certainty of evidence for the association between positive lymph node status and recurrence risk was rated as moderate. The random-effects model yielded a similar pooled OR of 2.86 (95% CI: 1.99–4.11, *p* < 0.01), further supporting this positive association. Heterogeneity among the studies was moderate, with an I² value of 30% (*p* = 0.21) (Figure 6), suggesting some variability in effect sizes across studies, though not statistically significant.

### 3.4 Sensitivity analysis

Sensitivity analyses were conducted using a leave-one-out approach for each pooled outcome. The pooled estimates remained stable throughout the analyses, and no single study significantly influenced the overall results, confirming the robustness of the meta-analysis findings.

## 4 Discussion

This meta-analysis aimed to evaluate the prognostic significance of tumor diameter, HPV status, depth of invasion, LVSI status, and positive lymph nodes in predicting recurrence risk in early-stage cervical cancer. Our findings demonstrate that tumor diameter >4 cm, depth of invasion >1/2, LVSI positivity, and positive lymph nodes are significantly associated with an increased risk of recurrence. According to the GRADE assessment, the certainty of evidence for these associations was generally moderate, except for HPV status and LVSI positivity, which were rated as low certainty. Our analysis showed that a tumor diameter greater than 4 cm was associated with a significantly higher risk of recurrence, corroborating previous studies that have linked larger tumor size with poorer prognosis (8, 17).

Larger tumors may have higher potential for micro metastasis and residual disease, thereby increasing the likelihood of recurrence (20, 21). This finding supports the inclusion of tumor size as a key factor in risk stratification models for early-stage cervical cancer, suggesting that patients with larger tumors may benefit from more intensive follow-up and possibly adjunctive therapy. This observation is consistent with prior studies demonstrating that increased tumor volume correlates with lymph vascular invasion, deeper stromal infiltration, and higher rates of lymph node metastasis (8–10). Moreover, tumors larger than 4 cm were independently associated with decreased disease-free survival and overall survival (22). Mechanistically, larger tumors may harbor more hypoxic regions, which can drive angiogenesis, epithelial-mesenchymal transition (EMT), and subsequent metastatic potential (23–25). Therefore, tumor diameter not only serves as a surrogate for tumor burden but also reflects underlying aggressive tumor biology. Recognizing tumor size >4 cm as a prognostic marker is crucial in guiding decisions regarding adjuvant radiotherapy or chemotherapy, particularly for patients with otherwise early-stage disease but unfavorable pathological features.

The relationship between HPV status and recurrence risk remains controversial. Although high-risk HPV types are known to be associated with cervical cancer development, our results did not find a consistent association between HPV positivity and recurrence risk (7, 26–28). This discrepancy may be due to the limited number of studies included in the analysis, the different methods used for HPV detection, and the possible influence of HPV-related immune responses on treatment outcomes. Further studies are warranted to clarify the role of HPV status in predicting recurrence, particularly in specific HPV subtypes, which may have varying impacts on prognosis. Depth of invasion and LVSI positivity were both strongly associated with an increased risk of recurrence in our analysis. Depth of invasion indicates the extent of tumor infiltration into the cervical stroma, which may reflect the tumor's aggressive nature and potential for spread (8). LVSI, on the other hand, represents the invasion of cancer cells into lymphatic or vascular spaces, serving as a marker for metastatic potential. These findings align with previous research suggesting that both factors are important predictors of recurrence and underscore the need to include them in risk stratification tools for early-stage cervical cancer (8, 9, 11, 15, 16). Clinicians may consider these factors when planning postoperative surveillance and assessing the need for adjuvant therapy. Positive lymph nodes were identified as one of the strongest predictors of recurrence, consistent with the role of lymphatic dissemination in cervical cancer progression (8, 9, 16). Patients with positive lymph nodes may have occult metastases that contribute to recurrence, even in cases treated at an early stage (29–31). This finding highlights the importance of thorough lymph node assessment during staging and suggests that patients with lymph node involvement may benefit from additional therapeutic interventions to reduce recurrence risk.

This meta-analysis has several limitations. First, although most included studies were of moderate to high quality based on the NOS, inherent biases from observational study designs could not be eliminated. Second, due to the limited number of studies per outcome (<10), assessment of publication bias was not reliable and therefore not formally conducted, as per Cochrane recommendations. Third, heterogeneity was substantial for some outcomes, particularly for HPV status and LVSI positivity, which may limit the generalizability of the findings. Lastly, the certainty of evidence for some outcomes, such as HPV positivity and LVSI, was low, emphasizing the need for cautious interpretation.

Future research should focus on large, multicenter, prospective studies with standardized methodologies to validate these findings. Particularly, further investigations into HPV subtypes and molecular biomarkers may enhance the predictive models for recurrence risk in early-stage cervical cancer.

## 5 Conclusion

This meta-analysis identified tumor diameter >4 cm, depth of invasion >1/2, LVSI positivity, and positive lymph node status as significant prognostic factors associated with an increased risk of recurrence in early-stage cervical cancer. The findings reinforce the importance of comprehensive pathological evaluation for risk stratification and postoperative management. By providing updated quantitative evidence, this study supports more individualized follow-up protocols and the consideration of adjuvant therapies for high-risk patients. Additionally, the inconclusive association between HPV status and recurrence highlights an important gap in the current knowledge, suggesting the need for further prospective studies focusing on HPV genotyping and molecular biomarkers. Overall, our results contribute to refining risk assessment strategies and guiding future research directions to improve outcomes for patients with early-stage cervical cancer.

## Data Availability

The original contributions presented in the study are included in the article/Supplementary Material, further inquiries can be directed to the corresponding author.
